# Biosynthesis of flower-shaped Au nanoclusters with EGCG and their application for drug delivery

**DOI:** 10.1186/s12951-018-0417-3

**Published:** 2018-11-13

**Authors:** Shichao Wu, Xiangrui Yang, Fanghong Luo, Ting Wu, Peilan Xu, Mingyuan Zou, Jianghua Yan

**Affiliations:** 1 Cancer Research Center, Medical College, Xiamen University, Xiamen, 361005 China; 2 Key Laboratory of Nanobiological Technology, Xiangya Hospital Central South University, Changsha, 410008 China; 3 Department of Chemistry, College of Chemistry and Chemical Engineering, Xiamen University, Xiamen, 361005 China

**Keywords:** Biosynthesis, Au nanoclusters, Drug delivery, Targeting, Dual drug

## Abstract

**Background:**

In the last decade, the biosynthesis of metal nanoparticles using organisms have received more and more considerations. However, the complex composition of organisms adds up to a great barrier for the characterization of biomolecules involved in the synthesis process and their biological mechanisms.

**Results:**

In this research, we biosynthesized a kind of flower-shaped Au nanoclusters (Au NCs) using one definite component—epigallocatechin gallate (EGCG), which was the main biomolecules of green tea polyphenols. Possessing good stability for 6 weeks and a size of 50 nm, the Au NCs might be a successful candidate for drug delivery. Hence, both methotrexate (MTX) and doxorubicin (DOX) were conjugated to the Au NCs through a bridge of cysteine (Cys). The introduction of MTX provided good targeting property for the Au NCs, and the conjugation of DOX provided good synergistic effect. Then, a novel kind of dual-drug loaded, tumor-targeted and highly efficient drug delivery system (Au-Cys-MTX/DOX NCs) for combination therapy was successfully prepared. The TEM of HeLa cells incubated with Au-Cys-MTX/DOX NCs indicated that the Au-Cys-MTX/DOX NCs could indeed enter and kill cancer cells. The Au-Cys-MTX/DOX NCs also possessed good targeting effect to the FA-receptors-overpressed cancer cells both in vitro and in vivo. Importantly, the Au-Cys-MTX/DOX NCs resulted in an excellent anticancer activity in vivo with negligible side effects.

**Conclusions:**

These results suggest that the biosynthesized Au-Cys-MTX/DOX NCs could be a potential carrier with highly efficient anticancer properties for tumor-targeted drug delivery.

**Electronic supplementary material:**

The online version of this article (10.1186/s12951-018-0417-3) contains supplementary material, which is available to authorized users.

## Background

In recent years, metal nanoparticles (NPs) have received considerable research interest due to their potential applications in catalysis [1, 2], electronics [3, 4], chemical sensing [5], optics [6], and biology [7, 8]. Among the metal NPs, gold NPs, with the properties of optical effect [9], heating [10, 11], and biocompatibility [12, 13], are receiving the greatest attention, mainly because of their broader application in biomedical science [14, 15]. Particularly, monolayer-protected Au NPs have recently emerged as an attractive candidate for delivering various therapeutic agents such as drugs, peptides, proteins, and nucleic acids to their targets [16, 17]. Since the monolayer could range from small organic compounds to macromolecules, the function and the property of Au NPs, such as the stability, targeting ability and drug loading capacity, could be largely improved in many ways, which makes Au NPs one of the most promising candidate for cancer treatment [18–20].

Although the property of Au NPs could be optimized by the monolayer, the nature of Au NPs, which mostly depends on the synthetic procedure, is of equal importance to the Au-based drug delivery system [21]. There are various synthetic methods of Au NPs, such as chemical reduction, biological synthesis, lithography, ultraviolet irradiation, and so on [22, 23]. In recent years, the biosynthesis of Au NPs using organisms, as an environmentally friendly method (green chemistry), has become a major focus of researchers [24, 25]. Without use of harsh, and toxic chemicals, the safety of Au NPs would be improved largely, which would increase their potential medical application, such as imaging, drug delivery, disinfection, and tissue repair [26]. Especially, the Au NPs produced by plants have been proved to be more various in shape and size in comparison with those produced by other organisms [27]. Although, the spherical morphology is still in the majority of the plants-synthetic Au NPs, a variety of non-sphere shapes began to appear. For example, triangular NPs have become another general Au NPs, which could be obtained with the reduction of *Aloe vera* [28], *Camellia sinensis* [29], *Cymbopogon flexuosus* [26], *Murraya koenigii* [30], pear fruit extract [31], and so on. In addition, hexagonal [32], rod-shaped [33], and meatball-like Au NPs [34] were also prepared successfully using plants extract. Unlike general chemical reduction, the plants extracts may act both as reducing and capping agents in the preparation process, which would make the Au NPs more stable. Moreover, the faster synthesis rate and lower cost make the plants-biosynthesis an ideal candidate for the large-scale production of Au NPs. Since the plants-synthetic Au NPs possess such a lot of pronounced properties, there must be extensive and promising applications in the future. However, mainly owing to the complexity and variety of the plants extract, the research about the plants-synthesis methods is still very limited, especially in regard to the detection and characterization of biomolecules involved in the synthesis process, which play a parameter role on understanding the biological mechanisms of the biosynthesis.

Tea extract has always been a nice candidate for the biosynthesis of metal NPs, who would possess good stability and uniformity [35–37]. Nevertheless, the biomolecules with the critical function are still not fully understood in the tea-synthesis process. It is commonly accepted that there might be at least two biomolecules participated in the process, which act as the reducing and capping agent, respectively. However, we found that epigallocatechin gallate (EGCG), the most abundant catechin in green tea, could play the role of reducing and capping agent at the same time. Hence, in this work, a kind of flower-shaped Au nanoclusters (Au NCs) with a size of 50 nm were biosynthesized using only one component—EGCG. It is amazing that the flower-shaped Au NCs possessed surprisingly good uniformity and stability, which could be kept for more than 6 weeks without aggregation. Since the NPs of 50 nm were proved to be internalized much more rapidly and efficiently by cancer cells than those of other sizes, we investigated the application of the flower-shaped Au NCs as drug deliver carriers. Both methotrexate (MTX) and doxorubicin (DOX) were conjugated to the Au NCs through a bridge of cysteine. With the targeting property of MTX and the synergistic effect of the both drugs, a novel kind of dual-drug loaded, tumor-targeted and high efficient drug delivery system (Au-Cys-MTX/DOX NCs) for combination therapy was successfully prepared. Then, the drug delivery property and the anticancer efficiency of the Au-Cys-MTX/DOX NCs were systematic evaluated in vitro and in vivo. The results illustrated that the Au-Cys-MTX/DOX NCs also showed excellent stability the same as that of Au NCs, which was one of the most essential properties for pharmaceutical application. With outstanding targeting property and enhanced anticancer efficiency, the Au-Cys-MTX/DOX NCs would become a promising anticancer drug delivery system for combination therapy.

## Materials and methods

### Materials

Hydrogen tetra chloroaurate (III) trihydrate (HAuCl_4_·4H_2_O, 99.9%) was purchased from Sinopharm Chemical Reagent Co. Ltd. Epigallocatechin gallate (EGCG, purity > 99%) was purchased from Hangzhou Gosun Biotechnologies Co., Ltd. MTX (purity > 99%) was purchased from Bio Basic Inc. Dicyclohexylcarbodiimide (DCC) and *N*-hydroxysuccinimide (NHS) were purchased from Sigma-Aldrich. DOX (purity > 99%), *S*-trityl-l-cysteine (TRCYS), trifluoroacetic acid (TFA), triisopropylsilane were purchased from Shanghai Macklin Biochemical Co., Ltd. DMEM/High glucose and fetal bovine serum (FBS) were from Gibco. All chemicals were of analytical grade and used as received without further purification. Ultrapure water (18.2 MΩ/cm) was used throughout the work.

### Animals and cell cultures

HeLa cells and HepG2 cells were frozen by medical college of Xiamen University. The complete growth medium was DMEM supplemented with FBS (10%) and penicillin/streptomycin (1%). The cells were cultivated in an incubator (Thermo Scientific) in the presence of 5% CO_2_ at 37 °C.

The BALB/C nude mice (5–6 weeks, 16–20 g) and BALB/C mice (5–6 weeks, 18–22 g) were purchased from Shanghai Laboratory Animal Center, Chinese Academy of Sciences. The tumor models were set up by injecting 1 × 10^6^ HeLa cells subcutaneously in the selected positions of the mice.

### Synthesis of flower-shaped Au nanoclusters (Au NCs)

Au NCs were synthesized by a microwave method. 0.25 mL of chloroauric acid solution (1 wt%) was added to a conical flask, followed by the addition of 23.25 mL of water. Then, 1.5 mL of EGCG (2.67 mg/mL) was introduced fleetly into the aqueous solution with vigorous stirring. The resulting mixture was immediately irradiated in a microwave oven (80 W) for 90 s when the yellowish-green color of Au NCs were appeared.

### Synthesis of TRCYS-MTX/DOX conjugations

MTX (45 mg), TEA (30 mg), DCC (10 mg) and NHS (5 mg) were added into DMF (2 mL) and stirred at rt for 1 h. Then TRCys (40 mg) was added into the solution and stirred for 6 h to obtain the TRCys-MTX conjugations. Next, DOX (54 mg) was added to the solution and stirred for another 6 h to obtain the TRCyts-MTX/DOX conjugations. While purifying, the mixture was treated with ultrapure water (6 mL) and acetic acid (0.01 M) until the pH reached 7.0, at which time the TRCys-MTX/DOX would precipitate out. Then the precipitate was washed with methanol for three times and lyophilized for 24 h to obtain the dry TRCys-MTX/DOX powders.

### Synthesis of dual-drug loaded Au nanoclusters (Au-Cys-MTX/DOX NCs)

TRCys-MTX/DOX (10 mg) were dissolved in DMF (2 mL). Then, trifluoroacetic acid (250 µL) and triisopropylsilane (50 µL) were added into the solution and stirred at 50 °C for 8 h to free the sulfydryl of Cys. Afterwards, freshly prepared Au NCs (5% wt, 2 mL) were added into the solution and stirred at rt for 4 h. At last, the suspicion was centrifuged at 8000 rpm for 7 min, and the precipitate was lyophilized for 24 h to obtain the dry Au-Cys-MTX/DOX NCs powders.

### Characterization

The structure of TRCys-MTX and TRCys-MTX/DOX were analyzed by H^1^NMR (AVANCE III 600 MHz). Morphology of the Au-Cys-MTX/DOX NCs and Au NCs was examined by SEM (UV-70) at 10 kV and TEM (Tecnai G2 Spirit) at 20 kV. The energy spectrum analysis of Au-Cys-MTX/DOX NCs and Au NCs was also examined by UV-70. The Size and zeta-potential values were determined by a Malvern Zetasizer Nano-ZS machine (Malvern Instruments, Malvern). Three parallel measurements were carried out to determine the average values. The content of Cys-MTX/DOX in Au-Cys-MTX/DOX NCs was determined by thermogravimetry analysis (SDT_Q 600) and ultraviolet spectrophotometry (Cary5000).

### In vitro drug release study

The in vitro drug release studies of Au-Cys-MTX/DOX NCs were performed using the dialysis technique. The Au-Cys-MTX/DOX NCs were dispersed in a PBS buffer solution (10 mL) and placed in a pre-swelled dialysis bag (MWCO = 3500 Da). The dialysis bag was then immersed in PBS (0.1 M, 150 mL, pH 7.4) and oscillated continuously in a shaker incubator (180 rpm) at 37 °C. All samples were assayed by high performance liquid chromatography (HPLC).

### In vitro intracellular distribution

The in vitro intracellular distribution of Au-Cys-MTX/DOX NCs were performed using a TEM (Tecnai G2 Spirit). HeLa cells were incubated in a 6-well plate with a density of 1 × 10^6^ cells per well. The cells were incubated at 37 °C and 5% CO_2_ for 24 h. The Au-Cys-MTX/DOX NCs (4 wt%) were added for 1 h, 2 h, 4 h, and 8 h. After incubation, the cells were washed six times with PBS and fixed with 4% paraformaldehyde for 2 h. And the cells were detached by trypsinization and rinsed with PBS for three times. Then, the samples were send to the school of life sciences of Xiamen University for further process. And the processed samples were examined by TEM (Tecnai G2 Spirit) at 20 kV.

### Confocal imaging of cells

MTX was labelled with Cy 5.5 via an amido bond to function as a fluorescent probe. The confocal imaging of cells were performed using a Leica laser scanning confocal microscope. Imaging of MTX-Cy 5.5 was carried out under the 673 nm laser excitation, and the emission was collected in the range of 700–750 nm. Imaging of DOX was carried out under the 480 nm laser excitation, and the emission was collected in the range of 550–600.

HeLa cells were incubated in 6-well plates with a density of 1 × 10^6^ cells per well. The cells were incubated with 5% CO_2_ at 37 °C for 24 h. The Au-Cys-MTX (-Cy 5.5)/DOX NCs, Au-Cys-MTX (-Cy 5.5) NCs, or Au-Cys-DOX NCs ([MTX] = 0.2 µmol/mL, [DOX] = 0.24 µmol/mL) were added to the cells for 4 h. After incubation, the cells were washed three times with PBS, fixed with 4% paraformaldehyde and stained with DAPI for 5 min. Subsequently, the cells were further washed thrice with PBS before confocal imaging.

Au-Cys-MTX (-Cy 5.5) NCs and Au-Cys-DOX NCs were synthesized via the same method with that of Au-Cys-MTX (-Cy 5.5)/DOX NCs without the use of DOX or MTX (-Cy 5.5).

### Cytotoxicity assays

The cytotoxicity of various particles mentioned before was determined by the WST-1 assay. Briefly, HeLa cells of exponential phase was plated in quintuplicate in a 96-well flat bottomed microplate at a density of 5000 cells per well and incubated for 24 h. Then, Au-Cys-MTX/DOX NCs, Au-Cys-MTX NCs, or Au-Cys-DOX NCs ([MTX] = 0.087, 0.154, 0.308, 0.616, 1.232, 2.464 µg/mL, [DOX] = 0.125, 0.25, 0.5, 1.0, 2.0, 4.0 µg/mL) were added to plate. After 24 h incubation, 10 µL per well WST-1 was added and kept for another 2 h, and the intensity of developed color was measured by micro plate reader (Dynastic MR 5000, USA) operating at 450 nm wavelength. Untreated cells were considered 100% viable.

To determine inhibitory drug concentrations (IC50) to stop 50% cell growth, dose response curves of MTX, DOX, and the nanoformations were performed. From the resulting curves of individual drug treatment and the dual drug nanoformations effects, the combination index (CI) for the nanoformations was calculated using the Chou–Talalay method:

$$\begin{aligned} {\text{CI}} & = \frac{{{\text{IC}}_{{50}} \;{\text{of}}\;{\text{DOX}}\;{\text{in Au-Cys-MTX}}/{\text{DOX}}\;{\text{NCs}}}}{{{\text{IC}}_{{50}} \;{\text{of}}\;{\text{DOX}}}} \hfill \\ & \quad + \frac{{{\text{IC}}_{{50}} \;{\text{of}}\;{\text{MTX}}\;{\text{in Au-Cys-MTX}}/{\text{DOX}}\;{\text{NCs}}}}{{{\text{IC}}_{{50}} \;{\text{of}}\;{\text{MTX}}}}\end{aligned}$$

In this analysis, synergy is defined when CI < 1. And the smaller the CI is, the stronger the synergy is.

### Biodistribution

For in vivo fluorescence imaging, Cy 5.5 was conjugated to the formations. MTX-Cy5.5, DOX-Cy5.5, Au-Cys-Cy 5.5 NCs, and Au-Cys-MTX/DOX-Cy5.5 NCs ([MTX] = 20 nmol/kg, [DOX] = 24 nmol/kg) were administered intravenously into the HeLa tumor-bearing nude mice via the tail veins. At predetermined time intervals, the mice were anesthetized with isoflurane (2.5%) and imaged with the Maestro in vivo imaging system (Cambridge Research & Instrumentation, Woburn, MA, USA). After 24 h, the mice were sacrificed, and the tumor and major organs (spleen, liver, kidney, lung, and heart) were excised, followed by washing the surface with 0.9% NaCl for the ex vivo imaging of Cy 5.5 fluorescence using a Maestro in vivo imaging system.

### Tumor inhibition in vivo

When the tumor volume of the HeLa tumor-bearing mice was approximately 60 mm^3^, the mice were randomly divided into 4 groups (10 mice per group), and treated by intravenous injection of 0.9% NaCl, the bulk MTX and DOX mixture, the mixture of Au-Cys-MTX NCs and Au-Cys-DOX NCs, and Au-Cys-MTX/DOX NCs ([MTX] = 10 nmol/kg, [DOX] = 12 nmol/kg) every 3 days for four times. The tumor volume and body weight were monitored every 3 days. The tumor volume was calculated by the following formula: tumor volume = 0.5 × length × width^2^. The highest and lowest data were discarded at last.

After 18 days, the mice were sacrificed and the tumors were excised and weighed. Next, the tumors were fixed in 4% paraformaldehyde overnight at 4 °C, embedded in paraffin, sectioned (4 μm), stained with hematoxylin and eosin (H&E), and examined using a digital microscopy system.

### Statistical analysis

The statistical significance of treatment outcomes was assessed using Student’s t-test (two-tailed); P < 0.05 was considered statistically significant in all analyses (95% confidence level).

## Results and discussion

### Synthesis of flower-shaped Au nanoclusters (Au NCs)

**Scheme 1:**
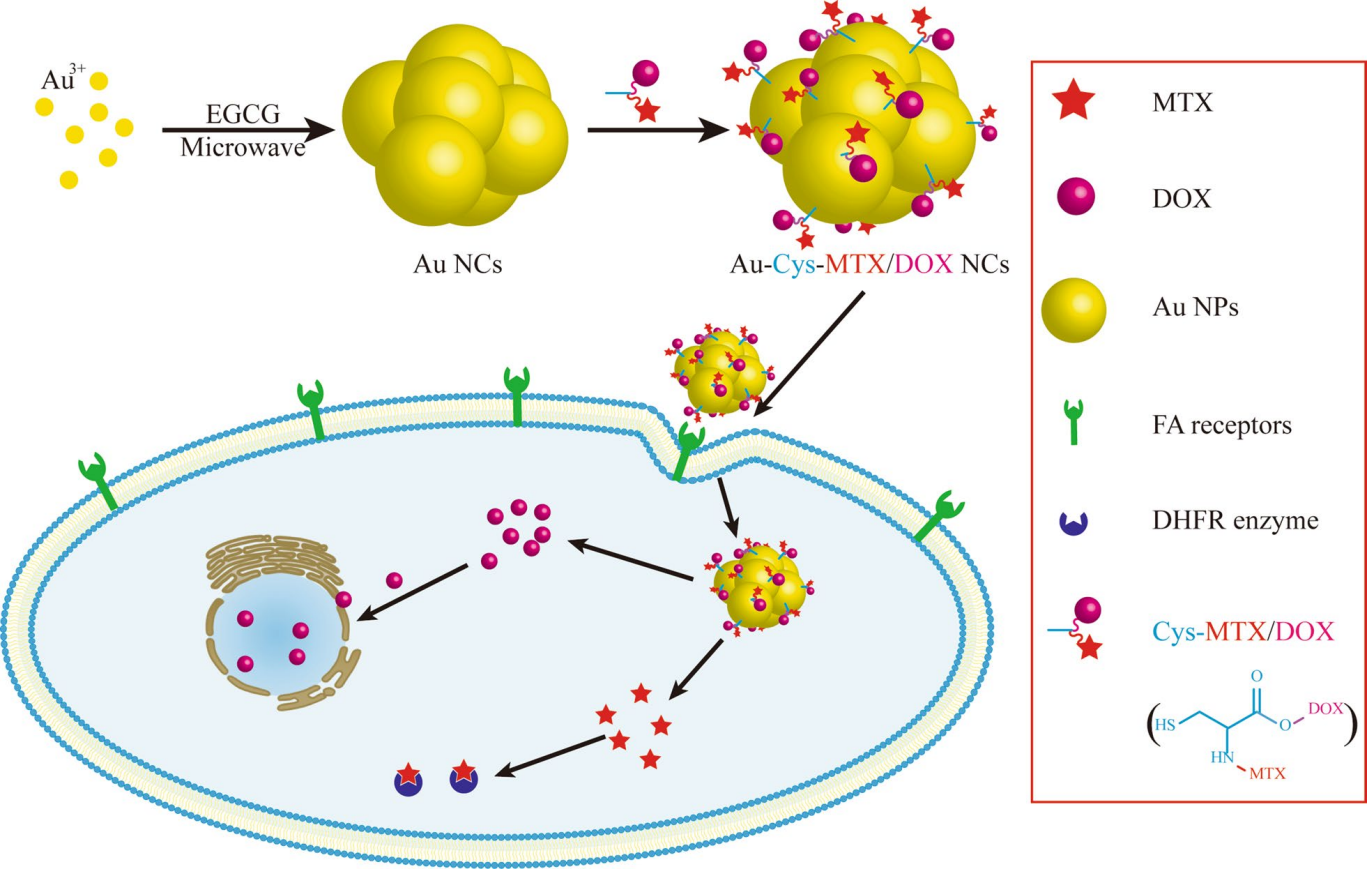
Illustration of the preparation of the Au-Cys-MTX/DOX NCs, and their targeted delivery to cancer cells and the simultaneous intracellular drug release of MTX and DOX

Different from other biosynthesis methods, a key feature of the experiments is the biosynthesis of flower-shaped Au nanoclusters (Au NCs) with only one component—EGCG. In short, the EGCG solution was introduced fleetly into the chloroauric acid solution under vigorous stirring conditions, followed by immediately irradiated in a microwave oven (see details in “Materials and methods”). Owing to the pronounced anti-oxidant property of EGCG, the chloroauric acid would be immediately deoxidized to gold atoms, which would precipitate out to form Au nanoparticles (Au NPs) and then aggregate to Au NCs (Scheme 1).

**Fig. 1:**
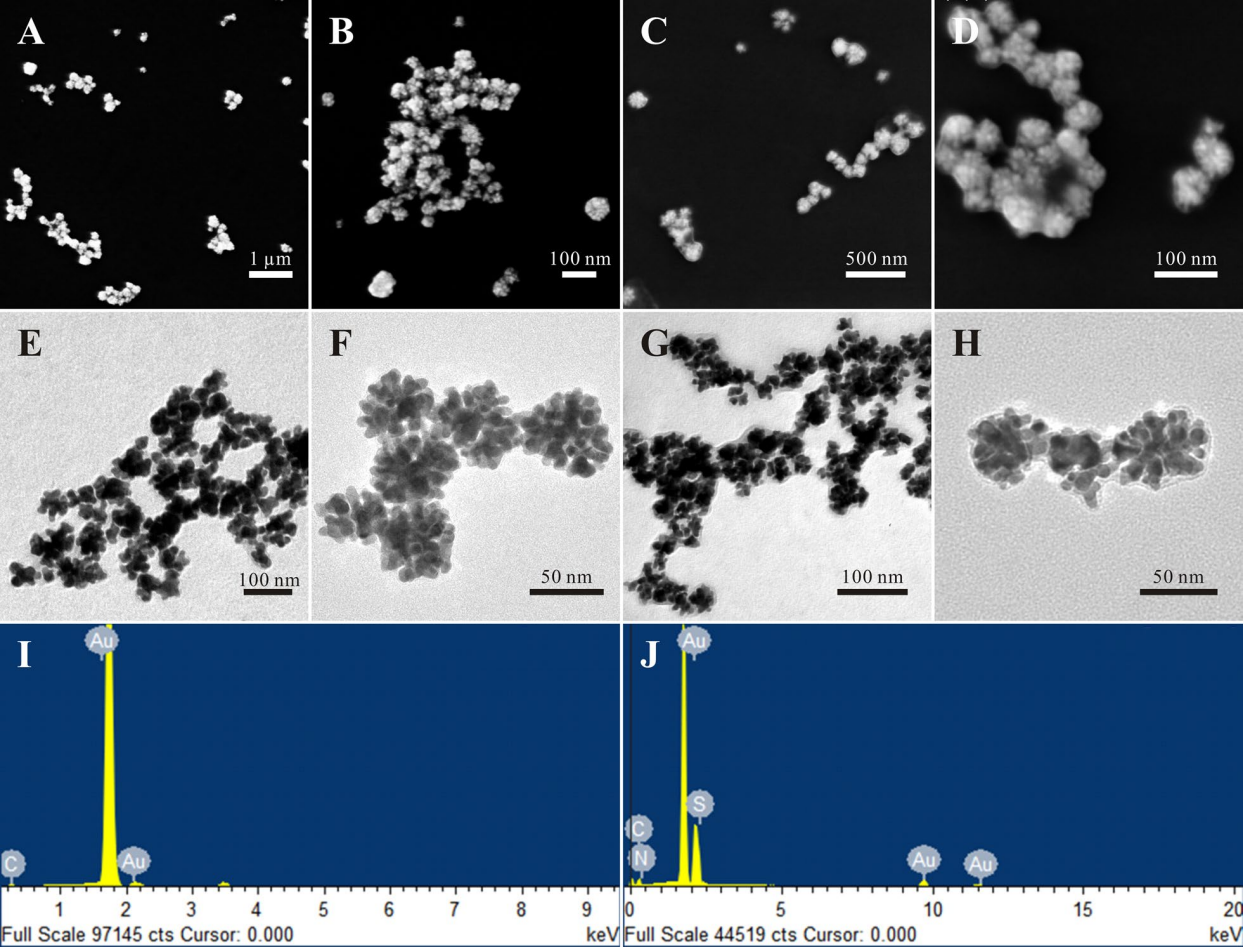
The SEM (**A**–**D**), TEM (**E**–**H**), and EDX (**I**, **J**) images of Au NCs (**A**, **B**, **E**, **F**, **I**) Au-Cys-MTX/DOX NCs (**C**, **D**, **G**, **H**, **J**)

The structural details of the Au NCs were well recorded by electron microscopes. SEM images and TEM images show that the Au NCs, with a uniform diameter of about 50 nm, look just like a flower and consist of several Au nanoparticles (Fig. 1A, B, E, F). In the high-resolution transmission electron microscopy image (Additional file 1: Figure S1A), the spacing of the crystallographic planes in the nanocrystal could be obviously distinguished and this spacing was 0.24 nm, which was in according with the distance between the (111) lattice planes of the Au crystal. The selected-area electron diffraction (SAED) pattern showed that the Au NCs were polycrystalline structure, which also indicated that they were formed from Au nanocrystals (Additional file 1: Figure S1B). Interestingly, the Au NCs, with a zeta potential of − 23.4 mV (Additional file 1: Figure S2A), exhibited especially high stability in water and phosphate buffered saline (PBS) even over 6 weeks after their synthesis (Additional file 1: Table S3). And it was more stable than other biosynthesized nanoparticles [38, 39], which could be stable for up to 4 weeks.

Since the nanoparticles could greatly improve the suboptimal pharmaceutical characters of the chemotherapeutics, the nanoparticle drug delivery system have received more and more attentions. And the study on cellular uptake of the particles have received great progress, although the influences are still not fully understood. According to previous works [16, 40–43], the cellular uptake shows a clear trend: the uptake of particles increases with the particle size of less than 50 nm, and decreases once the particles become larger. With the size of 50 nm, the Au NCs might show a highly enhanced effect in cancer cellular uptake. On account of this, we planned to conjugate dual anticancer drugs with synergistic effect to Au NCs, which might optimize the efficiency of drug delivery, and eventually benefit the cancer therapy.

### Synthesis of dual-drug loaded Au nanoclusters (Au-Cys-MTX/DOX NCs)

In Au-NPs-based drug delivery system, a sulfydryl-containing linker is always used to functionalize the Au NPs and then loaded with the model drug via acylation reactions [44–46]. However, the stabilizing EGCG on the surface of Au NCs might be destroyed by the acylation reaction, which would influence the stability of Au NCs. Hence, in this work, the dual-drug loaded, sulfydryl-containing group was firstly synthesized and then conjugated to Au NCs. As shown in Additional file 1: Formula S1, the dual-drug loaded Au NCs were synthesized in four steps: (1) TrCys-MTX synthesis: conjugation of MTX to TrCys via formation of an amido bond; (2) TrCys-MTX/DOX synthesis: conjugation of DOX to the TrCys-MTX via formation of another amido bond; (3) Cys-MTX/DOX synthesis: deprotection of the triphenylmethyl group; (4) Dual-drug loaded Au NCs (Au-Cys-MTX/DOX NCs): modification of Au NCs with Cys-MTX/DOX via Au–S bonds (the chemical equations are shown in Additional file 1: Formula S1). The H-nuclear magnetic resonance (H^1^NMR) was used to monitor the reaction process.

The formations of TrCys-MTX and TrCys-MTX/DOX were confirmed by the H^1^NMR spectrum, as shown in Additional file 1: Figure S4. While many peaks (8.58 and 4.79 ppm) corresponded with the group of MTX in the H^1^NMR spectrum of TrCys-MTX, the peak from 7.19 to 7.32 ppm was ascribed to the protons of triphenylmethyl groups (Additional file 1: Figure S4A). The result confirmed the formation of TrCys-MTX. In the H^1^NMR spectrum of TrCys-MTX/DOX (Additional file 1: Figure S4B), in addition to the characteristic peaks of TrCys-MTX, the peak at 3.90 ppm was observed due to the methoxyl protons of DOX. These results clearly indicated the formation of TrCys-MTX/DOX. Since the peaks at 4.79 ppm and 3.90 ppm ascribed to MTX and DOX, respectively, they were integrated to calculate the ratio of the dual drugs. The ratio of MTX to DOX was 1:1.2.

Since the H^1^NMR of Au-Cys-MTX/DOX NCs was very similar to that of Cys-MTX/DOX, the structure of Au-Cys-MTX/DOX NCs could not be confirmed by H^1^NMR. Hence, the energy dispersive spectrum (EDS) was employed to investigate the elementary compositions of Au NCs and Au-Cys-MTX/DOX NCs. Compared with the EDS of Au NCs, the appearance of elemental S and N in the EDS of Au-Cys-MTX/DOX NCs corroborated the existence of -Cys-MTX/DOX (Fig. 1I, J, Additional file 1: Tables S1, S2). This confirmed that the Au-Cys-MTX/DOX NCs was successfully synthesized.

To observe the morphology changes of Au NCs after modification, SEM and TEM were employed once again, and the results are shown in Fig. 1. The SEM and TEM images show that the Au-Cys-MTX/DOX NCs maintained the flower shape (Fig. 1C, D, G, H). However, when compared with Au NCs, there was an obvious change that a fine coating could be observed clearly on the surface of the Au-Cys-MTX/DOX NCs, which could be attributed to the conjugation of Cys-MTX/DOX on Au NCs. This also confirmed that the decoration of Au NCs was successful, which was in according with the result of EDS. Another evidence of the decoration was the slightly increased particle size and the decreased zeta potential (Additional file 1: Figures S2, S3).

Then the drug loading capacity of Au-Cys-MTX/DOX NCs was evaluated via thermogravimetric analysis (TGA) (Additional file 1: Figure S5). The conjugated drug loading ranged from 8.2 to 20.9 wt%, which were obtained by changing the ratio of Au NCs to Cys-MTX/DOX (Additional file 1: Table S3). Since the UV-absorption of Au NCs was much weaker than that of Cys-MTX/DOX, the UV-absorption course of Au-Cys-MTX/DOX NCs was similar to that of Cys-MTX/DOX. Meanwhile, the drug loading of Au-Cys-MTX/DOX NCs was verified via UV spectrophotometry at 290 nm (Additional file 1: Figure S6), and results were in according with those via TGA (Additional file 1: Table S3). Interestingly, the drug loading was proved to play a paramount role on the stability of Au-Cys-MTX/DOX NCs (Additional file 1: Table S3). While the Au NCs kept good stability for more than 6 weeks, the Au-Cys-MTX/DOX NCs with a drug loading of 8.2 wt% could only stabilize for less than 5 weeks. When the drug loading increased to 20.9 wt%, the Au-Cys-MTX/DOX NCs would aggregate in 3 days. And the decreased Zeta potential also predicted their increasingly weak stability (Additional file 1: Table S3). In consideration of the drug loading and the stability, the Au-Cys-MTX/DOX NCs with a drug loading of 13.4 wt% were chosen for the following evaluation.

### In vitro drug release study

The amido bond in the Au-Cys-MTX/DOX NCs allows the sustained release of DOX and MTX. The in vitro release studies of the Au-Cys-MTX/DOX NCs (13.4% content) were performed using a dialysis technique, alongside with bulk DOX or MTX powders. All samples were assayed by high performance liquid chromatography (HPLC). The release profiles are shown in Additional file 1: Figure S7. The free DOX was almost completely released within 8 h, while the bulk MTX need 18 h. This was owing to the different solubility of the dual drugs. Without the existence of protease, only small amount of drugs could be released from the Au-Cys-MTX/DOX NCs, which could be ascribed to the firmness of amido bond. Hence, the released drug would increase with the addition of protease, which could break amido bond. Compared with the bulk drug, the release of MTX from Au-Cys-MTX/DOX NCs exhibited a sustained and prolonged profile over a period of 48 h, just the same as that of DOX. The result also suggested the same form of both drugs in Au-Cys-MTX/DOX NCs.

### In vitro intracellular distribution

**Fig. 2:**
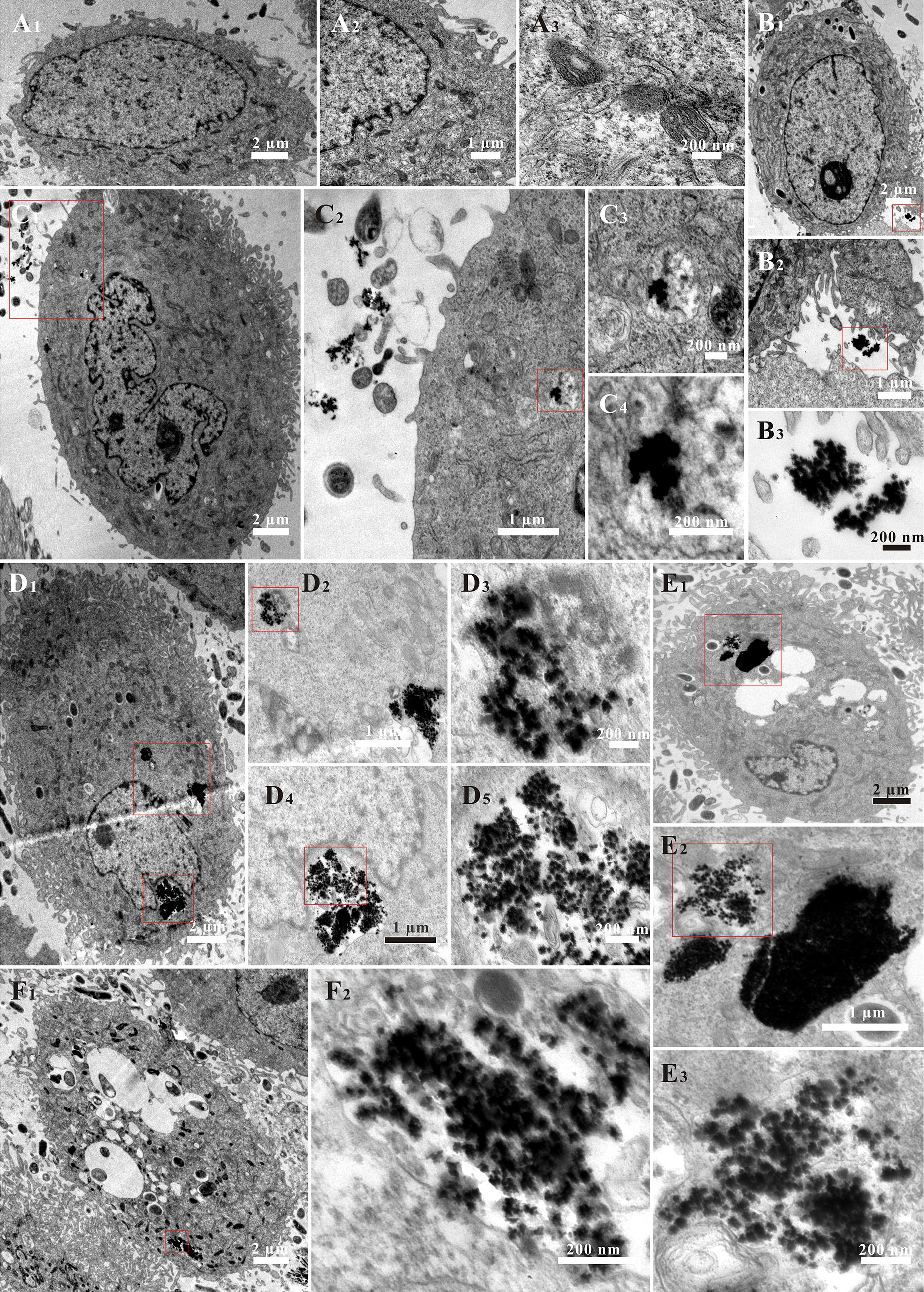
The TEM images of HeLa cells incubated with Au-Cys-MTX/DOX NCs at 37 °C for 0 h (**A**), 1 h (**B**, **C**), 2 h (**D**), 4 h (**E**), and 8 h (**F**)

The clear insight into the cellular internalization of the Au-Cys-MTX/DOX NCs by HeLa cells was gained by TEM (Fig. 2). HeLa cells were incubated with Au-Cys-MTX/DOX NCs for 1 h, 2 h, 4 h, and 8 h, and then the HeLa cells were analyzed using TEM. The HeLa cells without exposition to Au-Cys-MTX/DOX NCs were used for comparison. In the control group (Fig. 2A1–A3), the intact cell with several organelles could be observed, illustrating that the cells without incubation of Au-Cys-MTX/DOX NCs could keep functioning well during the course of the experiment. Although a small amount of Au-Cys-MTX/DOX NCs have entered into HeLa cells (Fig. 2C2–C4), a majority of Au-Cys-MTX/DOX NCs were still outside the cells after 1 h co-incubation (Fig. 2B1–B3 and C1, C2). When the incubation time increased to 2 h (Fig. 2D1–D5), the Au-Cys-MTX/DOX NCs in HeLa cells increased obviously. Several group of Au-Cys-MTX/DOX NCs could be seen in one cell and the flower shape was also faintly visible (Fig. 2D5). What’s more, the HeLa cells still kept regular cellular morphology at the first two sample times, which was just the same as that of the control group (Fig. 2A1, B1, C1 and D1). However, Au-Cys-MTX/DOX NCs continuously accumulated in the HeLa cells with the development of time (Fig. 2E1–E3), releasing more and more drugs which would kill the cells. Hence, several vacuolation appeared in the HeLa cells exposed to Au-Cys-MTX/DOX NCs for 4 h, illustrating that the cells were in an unhealthy state (Fig. 2E1). At 8 h, the vacuolation was more and the nucleus was hard to observe, demonstrating that the cell state became worse (Fig. 2F1, F2). The results proved that the Au-Cys-MTX/DOX NCs could enter and kill HeLa cells indeed.

### Confocal imaging of cells

**Fig. 3:**
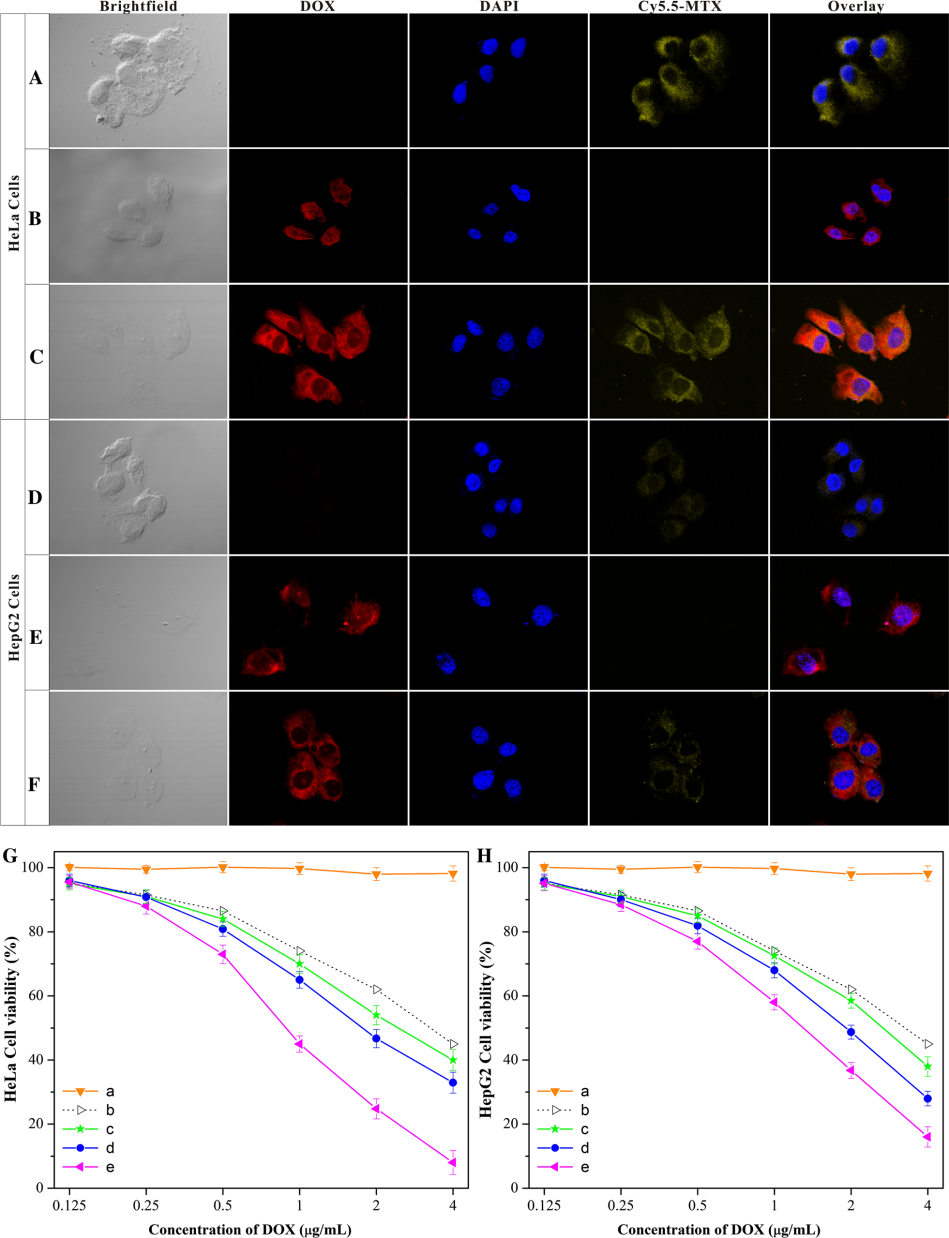
**A**–**F** Results of intracellular drug delivery in cancer cells, which were incubated for 4 h at 37 °C. **A**–**C**, CLSM images of HeLa cells incubated with Au-Cys-MTX NCs (**A**), Au-Cys-DOX NCs (**B**), Au-Cys-MTX/DOX NCs (**C**). MTX were pretreated with Cy 5.5 for imaging the MTX ingredient. The red and yellow fluorescent contrasts indicate the presence of DOX and MTX, respectively. **D**–**F**, CLSM images of HepG2 cells incubated with Au-Cys-MTX NCs (**D**), Au-Cys-DOX NCs (**E**), Au-Cys-MTX/DOX NCs (**F**). **G**, **H** In vitro cell viability of HeLa cells (**G**) or HepG2 cells (**H**) treated with the Au NCs (a), the theoretical value of bulk DOX and MTX (b), the mixture of MTX and DOX (c), the mixture of Au-Cys-DOX NCs and Au-Cys-MTX NCs (d), and Au-Cys-MTX/DOX NCs (e) after incubation of 24 h. P < 0.05

To obtain a more complete understanding of particle intracellular efficiency, confocal laser scanning microscopy (CLSM) was employed to monitor the Au-Cys-MTX/DOX NCs internalization. To access its cellular uptake, MTX was coupled with a fluorescent compound—cyanine 5.5 (Cy 5.5) beforehand for enhancing the visualization of MTX under CLSM. After 2 h incubation, both red and yellow fluorescence signals could been visualized in HeLa cells (Fig. 3C). It was indicated that Au-Cys-MTX (-Cy 5.5)/DOX NCs could deliver both drugs into HeLa cells, contributing to the synergistic anticancer treatment. Since MTX and DOX were both conjugated to the cysteine and then to the Au NCs, the fluorescence signals belong to the two drugs were overlapped largely at such a short incubation time − 2 h. To evaluate the targeting property of MTX, two kinds of single drug loaded nanoclusters (Au-Cys-MTX-Cy 5.5 NCs and Au-Cys-DOX NCs) were used as comparison. Just as was expected, an equally strong yellow fluorescence signal could be detected from the HeLa cells exposed to the Au-Cys-MTX-Cy 5.5 NCs, whereas a much weaker red fluorescence signal were collected when Au-Cys-DOX NCs was used (Fig. 3A, B). This difference illustrated that the targeting property of MTX could function very well in Au-Cys-MTX/DOX NCs, which would effectively enhance the cellular uptake. To access the role of FA receptor in the targeting process, HepG2 cells, absence of FA receptors, was used in the experiment. As shown in Fig. 3D–F, equally weak fluorescence signals were detected from the dual and single drug loaded nanoparticles, suggesting the absence of MTX-mediate targeting. The results indicated that the Au-Cys-MTX/DOX NCs would possess good targeting effect to the FA-receptors-overpressed cancer cells.

### Cytotoxicity assays

The killing ability of Au-Cys-MTX/DOX NCs to cancer cells was studied thereafter (Fig. 3G, H). The cytotoxicity was evaluated using the MTT assay with HeLa cells and HepG2 cells. The Au NCs were proved to be biocompatible at the concentration used in the cytotoxicity test. Firstly, the killing ability of the individual free drugs to the two kind of cells were tested, through which the theoretical value of the two combined drugs was figured out (Additional file 1: Figure S8). Then, the cytotoxicity of the physical mixture of MTX and DOX was tested, which was a little higher than the theoretical value, indicating the synergistic effect of both agents. And the mixture of Au-Cys-MTX NCs and Au-Cys-DOX NCs significantly showed higher cytotoxicity than that of the abovementioned physical mixture. This increase may be attributed to that the Au NCs might help to deliver the drug into cancer cells, especially to MTX, whose solubility was relatively poor. Moreover, Au-Cys-MTX/DOX NCs exhibited the highest cytotoxicity to the two kinds of cells. As to the HeLa cells, this further enhancement can be ascribed to the targeting property of MTX, which would increase DOX concentration in HeLa cells compared to Au-Cys-DOX NCs. However, as to the HepG2 cells, this was really an unexpected result to us, since the targeting property of MTX did not function owing to the absence of FA receptors. Although we repeated the experiment for many times, the results were just the same. This possible reason might be that the Au-Cys-MTX/DOX NCs possessed higher efficiency than the mixture of Au-Cys-MTX NCs and Au-Cys-DOX NCs in drug delivery. Although the concentrations of the dual drug were at the same level, the NCs taken up by the cells played the decisive role in killing the cells. When the single drug loaded NCs could only transport single drug into the cells at a time, the Au-Cys-MTX/DOX NCs would deliver the dual drugs into the cells simultaneously. In other words, to achieve the same effect, the number of NCs taken up by the cells in the group of the mixture of single drug loaded NCs should be twice as many as those of Au-Cys-MTX/DOX NCs. However, the rate of cellular uptake of the single drug loaded NCs might be less than two times of that of the Au-Cys-MTX/DOX NCs, leading to that the cytotoxicity of Au-Cys-MTX/DOX NCs to the HepG2 cells was higher than that of the mixture of the single drug loaded NCs. All these results indicated that the Au-Cys-MTX/DOX NCs would greatly enhance the synergetic effect of DOX and MTX. To give more direction on this enhancement, the combination index (CI), which could quantify the synergetic effect of multiple drugs, was calculated based on the Chou-Talalay equation [47, 48]. As shown in Table 1, the CI value of the physical mixture was less than 1, indicating the synergistic effect of MTX and DOX. What’s more, the CI value of Au-Cys-MTX/DOX NCs was much less than that of the physical mixture, which revealed the largely enhanced synergetic effect.

**Table 1:** The CI value of different formulations

Formulations	HeLa cells	HepG2 cells
MTX and DOX	0.48 ± 0.06	0.81 ± 0.11
Au-Cys-MTX NCs and Au-Cys-DOX NCs	0.34 ± 0.05	0.50 ± 0.07
Au-Cys-MTX/DOX NCs	0.17 ± 0.07	0.35 ± 0.06

### Biodistribution

**Fig. 4:**
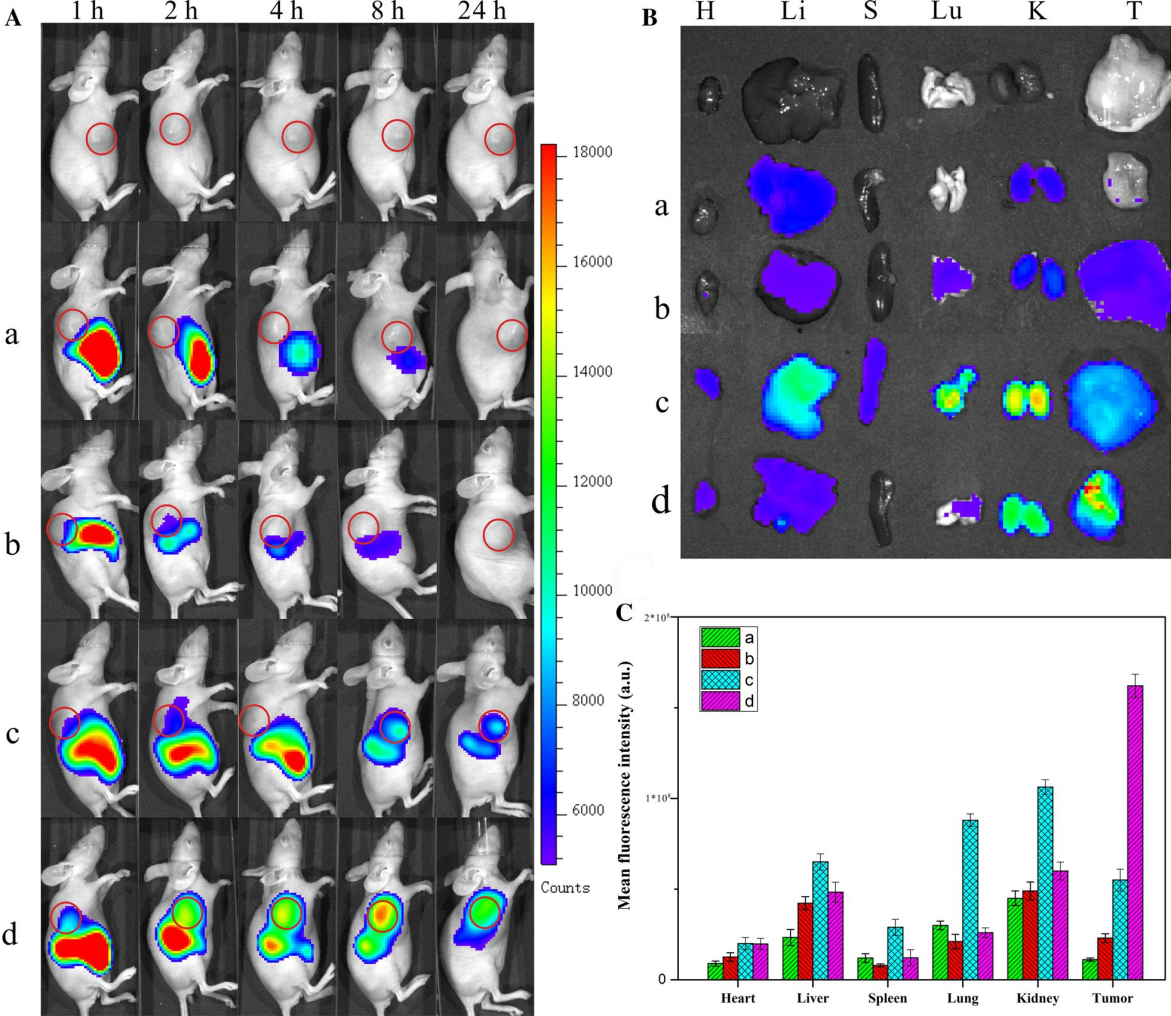
**A** Distribution and tumor accumulation of Cy 5.5-nanoparticles in HeLa tumor-bearing mice receiving intravenous injection of the indicated formulations. **B** Ex vivo fluorescence imaging of the tumor and normal tissues harvested from the euthanized HeLa tumor-bearing nude mice. The images were taken 24 h after the injection. H, Li, Lu, K, S, and T represent heart, liver, lung, kidney, spleen, and tumor, respectively. **C** Cy 5.5 fluorescence intensity in tumor tissues collected at 24 h following systemic injection. P < 0.05. (a) DOX-Cy 5.5, (b) MTX-Cy 5.5, (c) Au-Cys-Cy 5.5 NCs, (d) Au-Cys-MTX/DOX-Cy 5.5 NCs

The Au-Cys-MTX/DOX NCs mentioned beforehand were subsequently employed in tumor treatments on mice to access their efficacy in vivo. Firstly, the in vivo biodistributions of DOX, MTX, Au NCs, and the Au-Cys-MTX/DOX NCs were studied to evaluate their tumor targeting ability in vivo, which had been deemed as a crucial factor to evaluate their anticancer capability. Cy 5.5 was used as a near-infrared fluorescence probe to trace their biodistributions of the formations. After intravenously injected the formulations-Cy 5.5, the nude mice were imaged using the in vivo imaging system at predetermined time intervals. As shown in Fig. 4A, intense fluorescent signals were visualized at the liver areas in all the groups at the sample time of 1 h, indicating that the formulations-Cy 5.5 were first aggregated in the liver. However, while the free drug formulation (DOX-Cy 5.5 and MTX-Cy 5.5) were cleared quickly and no visible signals appeared at the tumor site, the NCs-formulation (Au-Cys-Cy 5.5 NCs, and the Au-Cys-MTX/DOX-Cy 5.5 NCs) revealed much longer half-life. More importantly, intense fluorescent signals were visualized at tumor site in the NCs-formulation group, suggesting their good targeting property. Especially in the group of Au-Cys-MTX/DOX-Cy 5.5 NCs, owing to the EPR effects and the pronounced targeting property of MTX on the surface, the intensity of the fluorescent signal at the tumor area increased gradually to a very high degree in the first 8 h, indicating the continuous and sustained accumulation of Au-Cys-MTX/DOX-Cy 5.5 NCs in tumors. As comparison, the signals in the liver of the same mouse decreased gradually during the course of the experiment. After 24 h, the mice were sacrificed to collect the fluorescent counts in tumor and normal tissues (Fig. 4B, C). The Au-Cys-MTX/DOX-Cy 5.5 NCs would significantly increase their accumulation in the tumor tissue compared with the other formulations. It was validated that the combination of MTX with the Au NCs would come into an excellent tumor targeting efficacy.

### Tumor inhibition in vivo

**Fig. 5:**
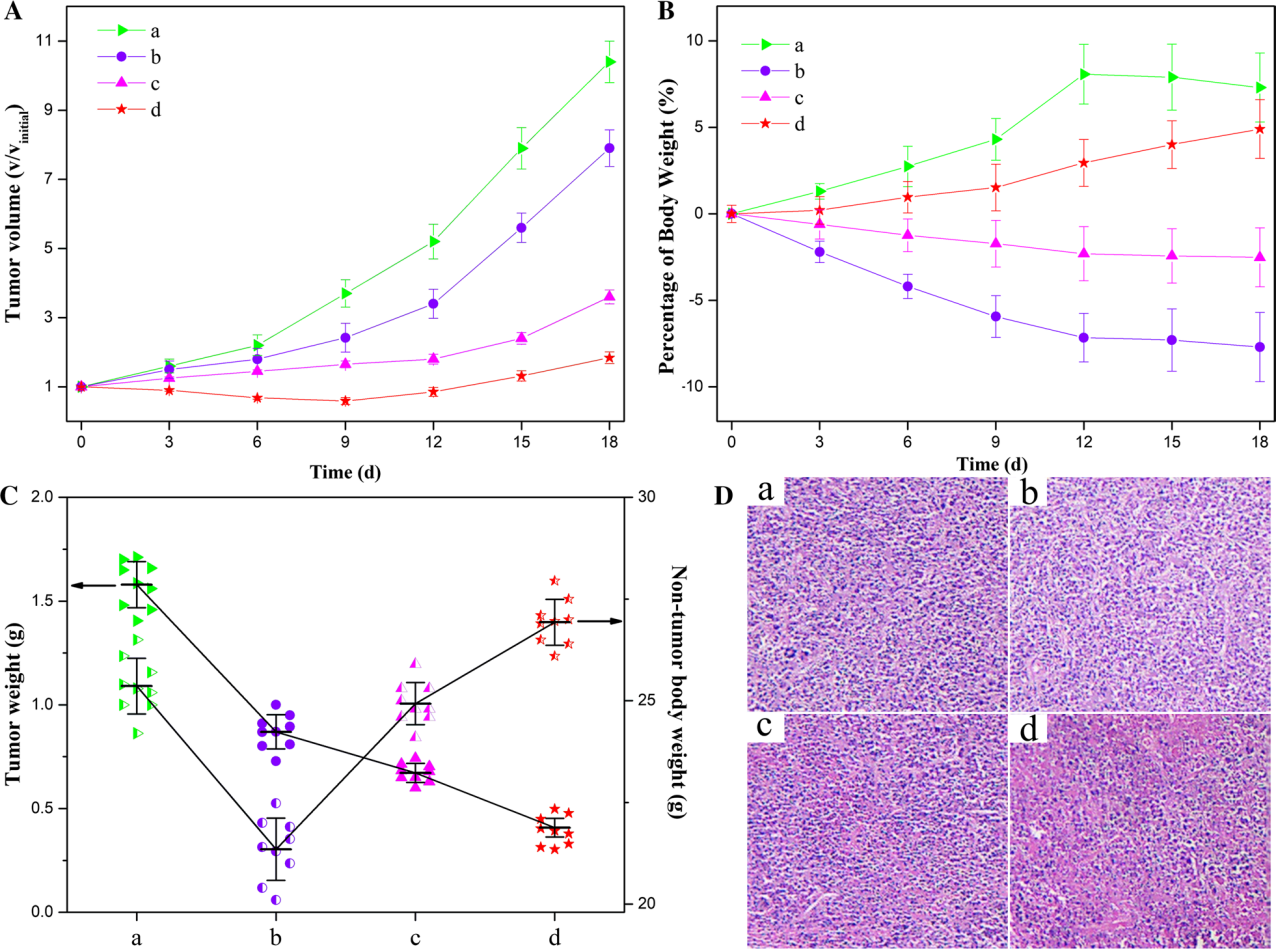
Anticancer effects of different formulations. **A** Volume change of tumor in mice during the treatment. **B** Weight change of the tumor-bearing mice during the treatment. **C** Weights of HeLa tumors and the non-tumor body weight after being treated by different formulations. **D** Histological section of the tumor of the mice after the treatment. (a) 0.9% NaCl aqueous solution, (b) the mixture of bulk DOX and MTX, (c) the mixture of Au-Cys-MTX NCs and Au-Cys-DOX NCs, and (d) Au-Cys-MTX/DOX NCs. All DOX-MTX formulations used the same concentration of DOX and MTX in mice bearing HeLa tumor. P < 0.05

Moreover, in vivo anticancer effects of the Au-Cys-MTX/DOX NCs were investigated by evaluating their efficacy of tumor inhibition. The mixture of free drug and the mixture of the single drug loaded NCs were used as control. Compared with the control injection of the 0.9% NaCl solution, all the formulations showed different levels of anticancer effects (Fig. 5). Bringing about the minimum tumor volume and weight, the Au-Cys-MTX/DOX NCs possessed the most pronounced inhibition effect (Fig. 5A, C). What’s more, the mice in the group showed gradually increased body weight and the maximum non-tumor body weight, indicating their better health conditions than those in other groups (Fig. 5B, C). An additional evidence of the enhanced anticancer effect of the Au-Cys-MTX/DOX NCs was shown in the histologic images (Fig. 5D). Compared to the other groups, the Au-Cys-MTX/DOX NCs led to the maximum necrotic regions, indicating their more outstanding anticancer efficacy. All these results stated that the Au-Cys-MTX/DOX NCs could effectively inhibit the tumor with hardly any side effect. As comparison, the physical mixture of DOX and MTX showed very bad anticancer effect as well as very serious side effect, coursing listlessness/laziness, severe body weight loss of mice (Fig. 5B) and minimum non-tumor body weight (Fig. 5C). As to the mixture of Au-Cys-MTX NCs and Au-Cys-DOX NCs, although their anticancer effect and the side effect have been high improved compared with the mixture of free drug, they were still nothing in comparison with those of Au-Cys-MTX/DOX NCs. Overall, the results clearly indicated that the Au-Cys-MTX/DOX NCs with the significant anticancer effect and low toxicity would greatly improve the efficacy of cancer therapy.

## Conclusion

In summary, the current study presents an environmentally friendly method using EGCG to obtain a flower-shaped Au nanoclusters with pronounced stability. Then as a carrier, the Au NCs were loaded with MTX and DOX for cancer therapy. The sustained and prolonged drug release property of the dual drug loaded Au NCs increases the acting time of the drug in vivo. The targeting property of MTX and the efficient-cellular-uptake size allow for enhanced targeting property to cancer cells. Plus the good synergistic effect of both drugs, the Au-Cys-MTX/DOX NCs possess excellent anticancer effect both in vivo and in vitro. The current study highlights the feasibility of biosynthesized Au nanoparticles for drug delivery. Particularly interestingly, biosynthesis method with one component opens a door for eco-friendly technique for production of well-characterized nanoparticles and provide a foundation for understanding their biological mechanisms.

## Additional file

**Additional file 1.** Formula, Additional tables and figures.
