# Supplementary material for: Biosynthesis of flower-shaped Au nanoclusters with EGCG and their application for drug delivery
Source: J Nanobiotechnology. 2018 Nov 13;16:90. doi: 10.1186/s12951-018-0417-3 (PMC6233264; doi:10.1186/s12951-018-0417-3)
Supplement: Supplementary file 1 — Additional file 1. Formula, Additional tables and figures. [file 12951_2018_417_MOESM1_ESM.docx]

**Additional Information**

**Biosynthesis of Flower-shaped Au Nanoclusters with EGCG and their Application for Drug Delivery**

Shichao Wu, Xiangrui Yang, Fanghong Luo, Ting Wu, Peilan Xu, Mingyuan Zou, ＆ Jianghua Yan.

**The file includes**

1. Formula S1
2. Table S1-3
3. Figure S1-8

**Formula S1.** The formal chemical equations of the synthetic procedure of Au-Cys-MTX/DOX NCs


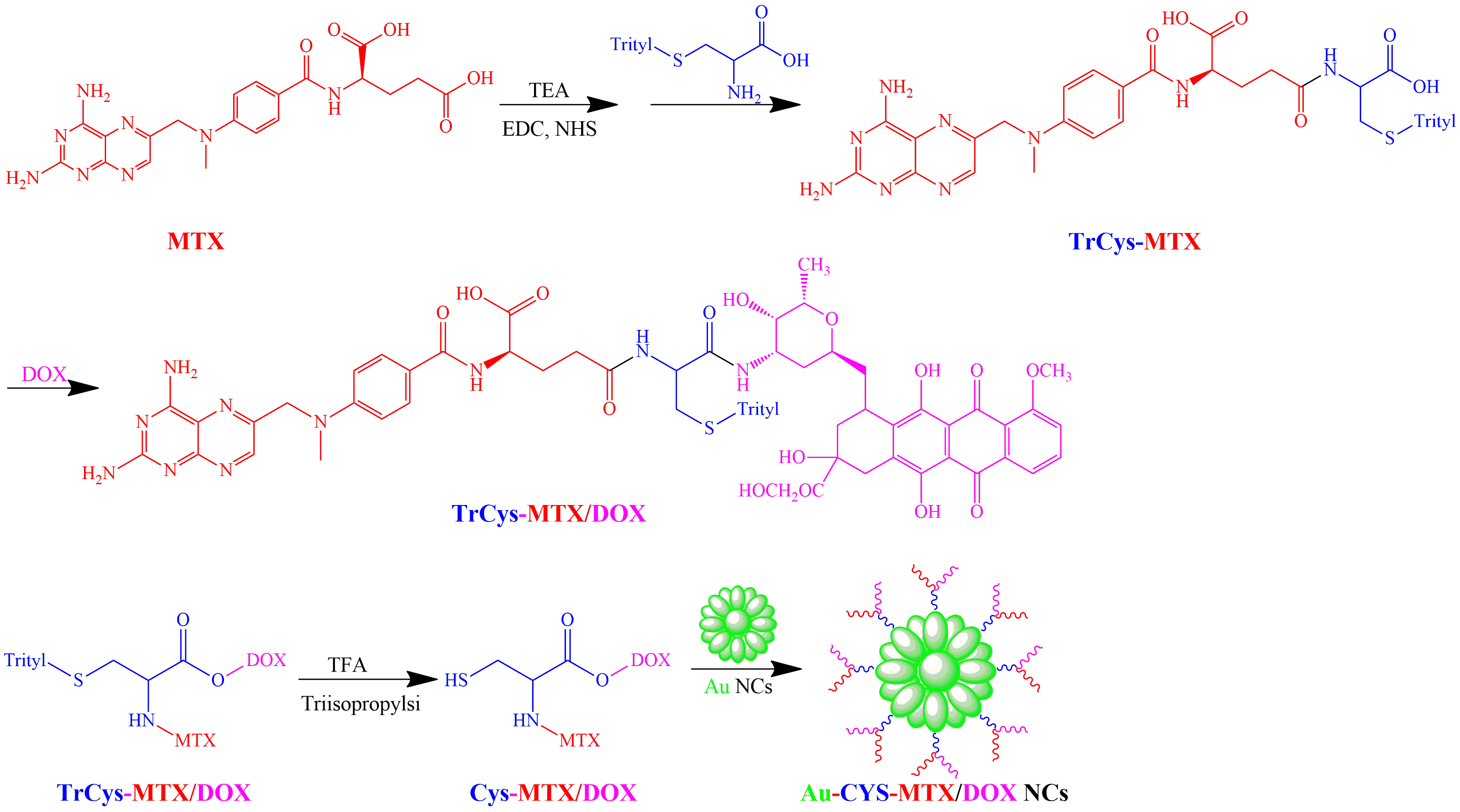


Table S1. The Element composition of Au NCs by EDS

| Element | Weight% | Atomic% |
| --- | --- | --- |
| C K | 4.38 | 27.07 |
| O K | 8.67 | 40.20 |
| N K | 0.00 | 0.00 |
| S K | 0.00 | 0.00 |
| Au K | 86.95 | 32.74 |
| Totals | 100 |  |

Table S2. The Element composition of Au-Cys-MTX/DOX NCs by EDS

| Element | Weight% | Atomic% |
| --- | --- | --- |
| C K | 6.31 | 20.68 |
| O K | 19.35 | 50.94 |
| N K | 6.99 | 16.53 |
| S K | 3.36 | 6.59 |
| Au K | 63.99 | 12.80 |
| Totals | 100 |  |

Table S3. The Drug loading, Zeta potential and stability of Au-Cys-MTX/DOX NCs

| Drug loading (TGA, wt%) | Drug loading (UV, wt%) | Zeta potential(mv) | Stability(d) |
| --- | --- | --- | --- |
| 0 | 0 | -25.7 ± 1.2 | 43.4 ± 4.2 |
| 8.2 ± 1.2 | 8.6 ± 1.6 | -21.4 ± 1.0 | 33.8 ± 5.6 |
| 13.4 ± 1.1 | 13.9 ± 1.5 | -20.3 ± 0.6 | 27.9 ± 6.1 |
| 17.1 ± 1.5 | 17.9 ± 1.9 | -15.6 ± 0.7 | 15.4 ± 3.8 |
| 20.9 ± 2.4 | 21.3 ± 2.8 | -10.5 ± 0.9 | 3.4 ± 2.2 |


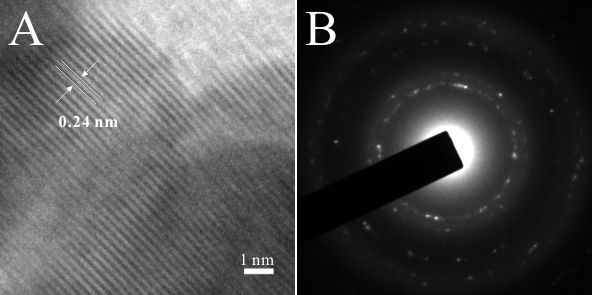


**Figure S1.** HRTEM image (A) and the SAED pattern (B) of Au NCs.


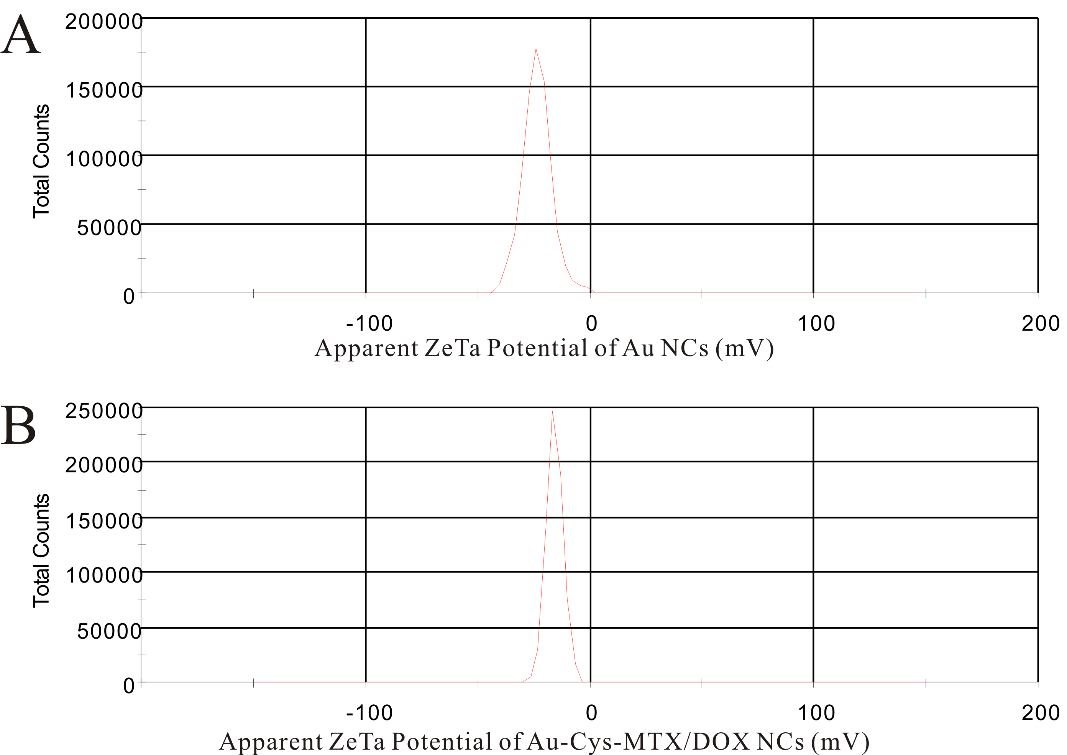


**Figure S2.** The Zeta potential of Au NCs (A) and Au-Cys-MTX/DOX NCs (B).





**Figure S3.** The Size distributions of Au NCs and Au-Cys-MTX/DOX NCs.


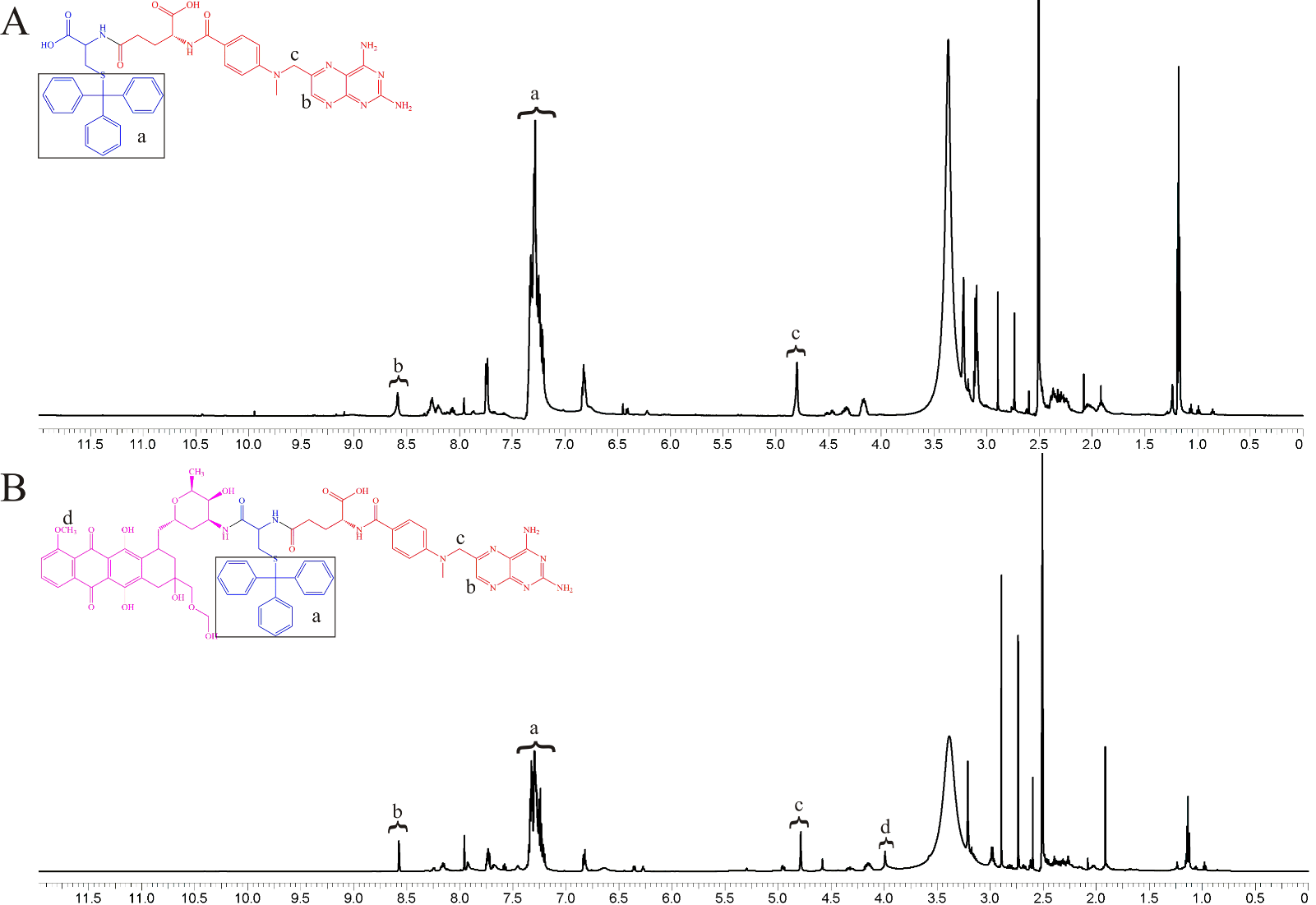


**Figure S4.** The H^1^NMR of TRCys-MTX (A) and TRCys-MTX/DOX (B).





**Figure S5.** TGA curves Au NCs (a) and Au-Cys-MTX/DOX NCs (b-e) with different drug loading.





**Figure S6.** UV/Vis absorbance spectras of Cys-MTX/DOX (a) and Au-Cys-MTX/DOX NCs (b).


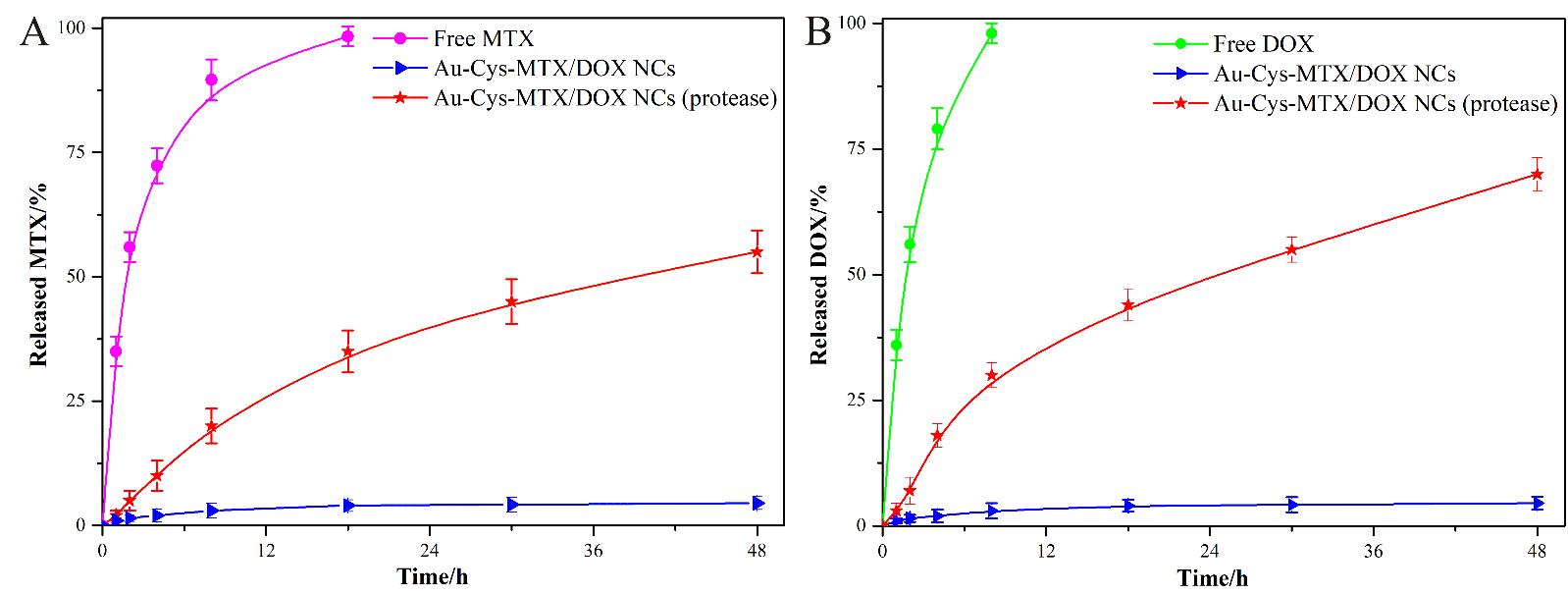


**Figure S7.** *In vitro* release profiles of free MTX, free DOX, and Au-Cys-MTX/DOX NCs.


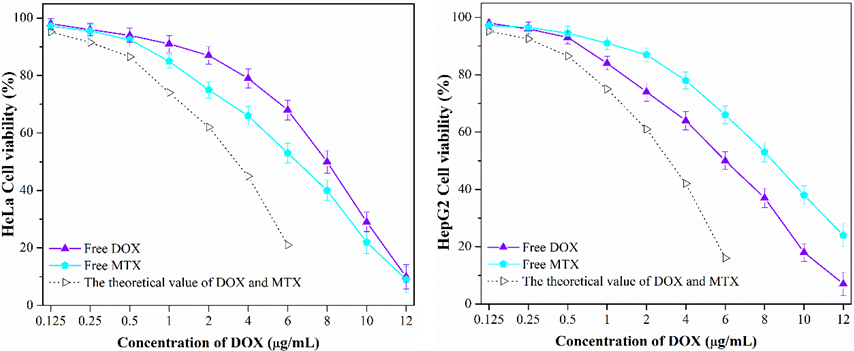


**Figure S8.** *In vitro* cell viability of HeLa cells or HepG2 cells treated with the free MTX, free DOX, and the theoretical value of bulk DOX and MTX after incubation of 24 h.
